# Biophysical and In Silico Studies of the Interaction between the Anti-Viral Agents Acyclovir and Penciclovir, and Human Serum Albumin

**DOI:** 10.3390/molecules22111906

**Published:** 2017-11-05

**Authors:** Ali S. Abdelhameed, Ahmed H. Bakheit, Fahad M. Almutairi, Haitham AlRabiah, Adnan A. Kadi

**Affiliations:** 1 Department of Pharmaceutical Chemistry, College of Pharmacy, King Saud University, P.O. Box 2457, Riyadh 11451, Saudi Arabia; abujazz76@gmail.com (A.H.B.); halrabiah@ksu.edu.sa (H.A.); akadi@ksu.edu.sa (A.A.K.); 2 Department of Chemistry, Faculty of Science and Technology, El-Neelain University, P.O. Box 12702, Khartoum 11121, Sudan; 3 Department of Biochemistry Faculty of Science, University of Tabuk, P.O. Box 741, Tabuk 71491, Saudi Arabia; falrabae@ut.edu.sa

**Keywords:** acyclovir, penciclovir, human serum albumin, spectroscopic techniques, in silico study

## Abstract

Acyclovir (ACV) and penciclovir (PNV) have been commonly used during the last few decades as potent antiviral agents, especially for the treatment of herpes virus infections. In the present research their binding properties with human serum albumin (HSA) were studied using different advanced spectroscopic and in-silico methods. The interactions between ACV/PNV and HSA at the three investigated temperatures revealed a static type of binding. Extraction of the thermodynamic parameters of the ACV-HSA and PNV-HSA systems from the measured spectrofluorimetric data demonstrated spontaneous interactions with an enthalpy change (∆*H*^0^) of −1.79 ± 0.29 and −4.47 ± 0.51 kJ·mol^−1^ for ACV and PNV, respectively. The entropy change (∆*S*^0^) of 79.40 ± 0.95 and 69.95 ± 1.69 J·mol^−1^·K^−1^ for ACV and PNV, respectively, hence supported a potential contribution of electrostatic binding forces to the ACV-HSA and PNV-HSA systems. Putative binding of ACV/PNV to HSA, using previously reported site markers, showed that ACV/PNV were bound to HSA within subdomains IIA and IIIA (Sudlow sites I and II). Further confirmation was obtained through molecular docking studies of ACV-HSA and PNV-HSA binding, which confirmed the binding site of ACV/PNV with the most stable configurations of ACV/PNV within the HSA. These ACV/PNV conformers were shown to have free energies of −25.61 and −22.01 kJ·mol^−1^ for ACV within the HSA sites I and II and −22.97 and −26.53 kJ·mol^−1^ for PNV in HSA sites I and II, with hydrogen bonding and electrostatic forces being the main binding forces in such conformers.

## 1. Introduction

**Figure 1:**
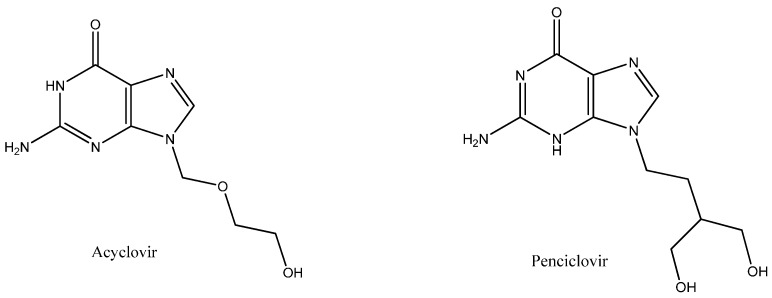
Chemical structures of acyclovir (ACV) and penciclovir (PNV).

Acyclovir (ACV), an acyclic analog of the innate nucleoside 2-deoxyguanosine (Figure 1), is very effective against a number of herpes groups in DNA viruses [1,2]. For over 3 decades, ACV has been considered a very potent and standard drug for the treatment of HSV-1 and HSV-2 (Herpes simplex virus 1 and 2); it is also effective against some other viral infections such as the varicella–zoster virus. Additionally, ACV protects against cytomegalovirus in immunosuppressed patients undergoing organ transplantation [3,4]. There are many structural analogues of ACV; one of them is penciclovir (PNV) (Figure 1). PNV is also effective against the members of the herpes virus family [5]. Some studies have shown that PNV exerts the same effect on herpes viruses as ACV, but at a lower dose than ACV [6]. Both ACV and PNV target viral DNA and prevent viral DNA synthesis and ultimately virus replication [6,7]. 

ACV and PNV both enter virally-infected as well as non-infected cells, but they are phosphorylated only in virus-infected cells and the enzyme required for the phosphorylation is thymidine kinase, encoded by viruses. This property of both the drugs makes them highly specific antiviral drugs [8,9].

The bioavailability of orally administered acyclovir is 15–30% [10], whilst it is 70–80% for penciclovir [11]. Studies of the binding properties of ACV and PNV as well as other drugs with serum albumin can provide a clue towards pharmacological properties of those drugs. Drugs’ pharmacokinetic and pharmacodynamic properties depend on the binding affinity of serum albumin with the drugs [12]. Human serum albumin (HSA) is the major protein in blood plasma and accounts for almost 60% of total plasma protein content [13]. It is also the chief transporting protein for ligands; it has the ability to bind with a wide range of small molecules including drugs [14]. The present investigation is constructed to thoroughly examine the binding of ACV and PNV with human serum albumin. Two earlier studies were published on the binding of ACV to BSA (bovine serum albumin), either alone [15] or in the presence of carbon nanotubes [16]. This study mainly utilized the ACV and PNV-induced fluorescence quenching of HSA to determine the mechanisms of ACV-HSA/PNV-HSA interactions and the various binding parameters. Moreover, a UV-Vis spectral analysis and molecular docking were used to confirm and analyze ACV-HSA and PNV-HSA interactions in-silico.

## 2. Results and Discussion

### 2.1. Measurements of the Fluorescence Emission Intensity

**Figure 2:**
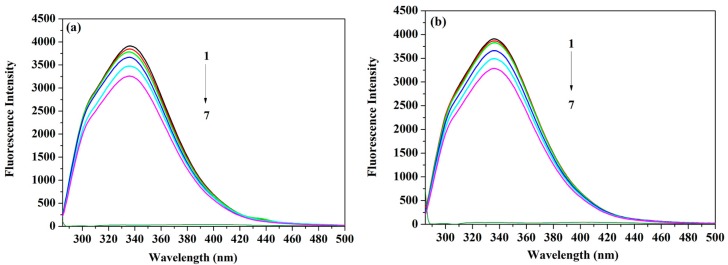
Representative spectra of (**a**) HSA (human serum albumin)-ACV (0, 3.5, 7, 15, 25 and 35 μM) and ACV only (25 μM) (**b**) HSA-PNV (0, 3.5, 8.0, 17, 28 and 35 μM) and PNV only (28 μM), shown as peaks 1–7 for each drug at temperature 298 K.

The efficiency of the fluorescence quenching approach as a technique used to inspect the protein binding process to a range of ligands inclusive of drugs has been previously established [17,18,19,20]. A wide range of binding information (e.g., strength and mechanism of binding, thermodynamic characteristics of the protein) can be obtained through these quenching studies [17,18]. In the same context, examining serum albumin’s binding to the different drugs can offer a thorough understanding of the drug journey inside the body [21,22]. Quenching of the fluorescence intensity can be due to either dynamic and static interaction, or both. The diffusion of molecules in the solution is responsible for the dynamic quenching, while the formation of a ground-state complex is the reason behind the static type [19]. Hence, under the experimental conditions described earlier, the HSA fluorescence intensity was quenched by the steady addition of ACV/PNV (Figure 2a,b) with no shift in the emission wavelength/peak shape. These fluorescence intensity determinations were carried out at temperatures of 288 K, 298 K and 309 K. Figure 2a and Figure 2b also demonstrate that both ACV and PNV possess negligible native fluorescence following their excitation at the same wavelength as the protein (i.e., λ_ex_ = 280 nm). Interpretation of such data was executed with the aid of the well-known Stern–Volmer (Equation (1)) [23] and Lineweaver–Burk equations (Equation (2)) [24], which describe dynamic and static mechanisms.

(1) F0F=1+KSVCQ=1+Kqτ0CQ

(2) $${\left(F_{0}-F\right)}^{-1}=\;F_{0}{}^{-1}+K_{LB}{}^{-1}F_{0}{}^{-1}C_{Q}{}^{-1}$$

In these equations, *F* and *F*_0_ stand for the intensity maximum of the HSA fluorescence emission, in the presence and absence of ACV/PNV, respectively. *C_Q_* represents the individual ligand concentration (ACV/PNV), *K_SV_* and *K_LB_* are the Stern–Volmer and Lineweaver–Burk constants, respectively. *K*_q_ represents the quenching rate constant and *τ*_0_ is the unquenched lifetime of the protein, which was set as 2.7 × 10^−9^ s^−1^ [19]. 

**Figure 3:**
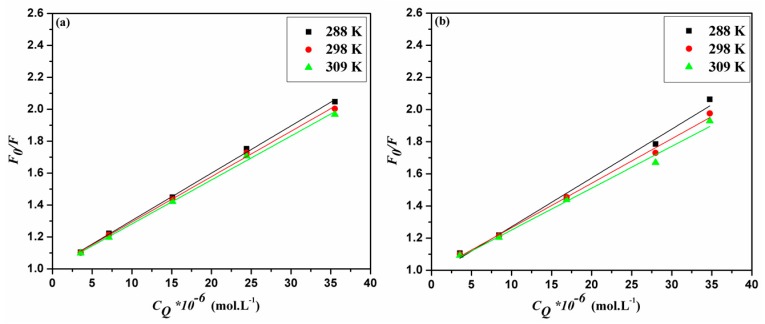

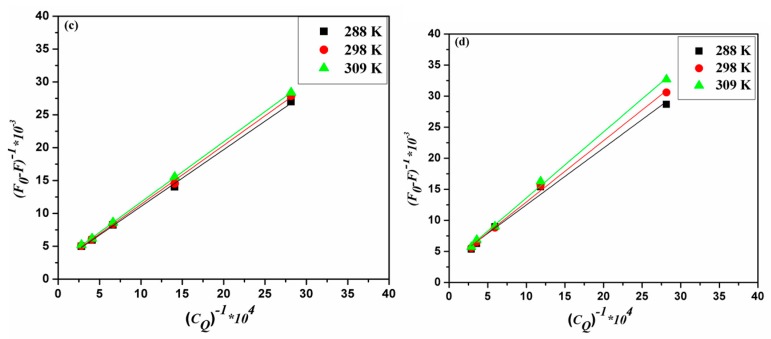
Stern–Volmer plots for (**a**) ACV-HSA and (**b**) PNV-HSA systems and Lineweaver–Burk plots for (**c**) ACV-HSA and (**d**) PNV-HSA systems.

The analysis of the emission data generated two linear plots for Stern–Volmer and Lineweaver–Burk relationships (Figure 3a,c for ACV and Figure 3b,d for PNV). Such plots may propose that static interactions are taking place in both cases of ACV and PNV with the HSA. This proposed static type of quenching was further promoted by the changes in Stern–Volmer and Lineweaver–Burk constants, in correlation with the change in temperature (Table 1). Both static and dynamic pathways of quenching can be differentiated based on their distinct dependency on temperature change, as the higher the temperature, the higher the Stern–Volmer and Lineweaver–Burk constants for the dynamic quenching, which is in contrast to the temperature effect for the static type. Consistent with the static quenching assumption, the bimolecular quenching rate constant (*K_q_*) values (Table 1) were estimated using Equation (3), and shown to be greater than the maximum value for a dynamic quenching process of 2 × 10^10^ L·M^−1^·s^−1^ [19].

(3) Kq=KSVτ0

### 2.2. Binding Mode and Binding Sites

**Figure 4:**
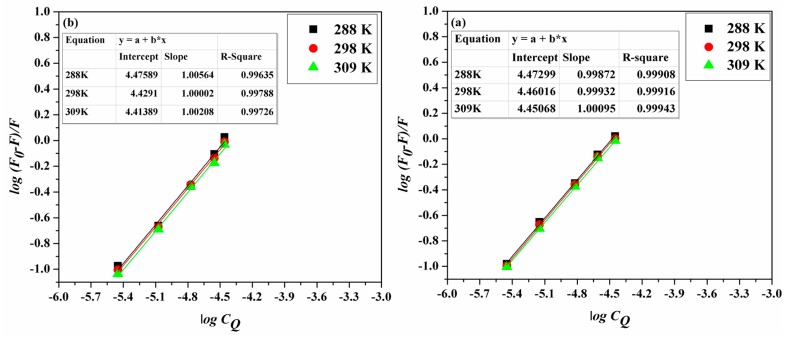
The double log plots for (**a**) ACV-HSA and (**b**) PNV-HSA systems at the studied temperatures.

The binding ability of the drug and its regulatory effect on the stability of the proteins helps to manage the therapeutic importance of the drug to some extent and therefore, it is very significant in studying the binding constant for the drug–protein interaction [25,26]. Hence, the recorded HSA fluorescence quenching data can be also manipulated using Equation (4), assuming a static binding and that the free and bound molecules are almost equal [27].

(4) $$\log\left(\frac{F_{0}-F}{F}\right)=\log K+n\log C_{Q}$$

where *K* represents the binding constant and *n* is the number of binding sites on the protein. Linearized plots of log(*F*_0_ − *F)/F* and log*C_Q_* (Figure 4a,b) permitted the estimation of *K* and *n* (Table 2). Table 2 shows that the *K* values comply with the temperature dependence role for the static and dynamic interactions—as described earlier—with both Stern–Volmer and Lineweaver–Burk constants.

### 2.3. Thermodynamic Parameters and Nature of the Binding Forces 

**Figure 5:**
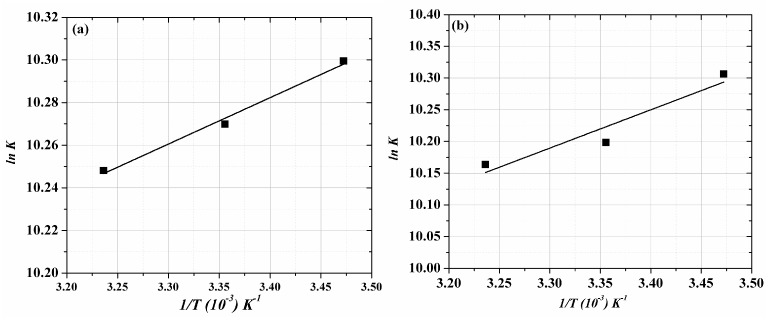
Van’t Hoff plots for (**a**) ACV-HSA and (**b**) PNV-HSA complexes.

Based on the earlier-discussed binding data, it can be observed that the ACV/PNV binding to HSA is greatly affected by the change in temperature, hence, the thermodynamic parameters can be derived from the fluorescence data. These parameters can further help to identify the binding forces involved in the ACV-HSA/PNV-HSA interactions. Such forces may include hydrogen bonding, hydrophobic, van der Waals or electrostatic interactions. Former reports have recognized the various forces in a protein/ligand binding in line with the determined thermodynamic parameters [28,29,30]. Such measurements have revealed three potential properties, of which a hydrophobic interaction is claimed if there are positive entropy change (∆*S*^0^) and enthalpy change (∆*H*^0^) values. Hydrogen bonding and/or van der Waals forces could be the driving forces when negative ∆*H*^0^ and ∆*S*^0^ are observed, and electrostatic forces are deemed responsible when negative ∆*H*^0^ and positive ∆*S*^0^ are present. Hence, to estimate the thermodynamic parameters involved in the ACV/PNV and HSA interactions, the following Equations (5) and (6) were used.

(5) $$\ln K=\frac{-\Delta H^{0}}{RT}+\frac{\Delta S^{0}}{R}$$

(6) $$\Delta G^{0}=\Delta H^{0}-T\cdot \Delta S^{0}$$

where *R* is the gas constant, *K* is the binding constant and *T* is the temperature (Kelvins). The following, plot of ln *K* and 1*/T* (Figure 5a,b) shows the thermodynamic values of the ACV/PNV-HSA interaction (Table 2). The results listed in Table 2 show that ACV and PNV spontaneously interact with the HSA through electrostatic forces. However, this finding cannot confidently exclude the role of hydrogen bonding, as the negative ∆*H*^0^ values in such interactions should appear very close to zero, to claim there are exclusively electrostatic interactions, which is not the case for ACV or PNV [31]. Moreover, earlier reports showed that a negative Δ*H*^0^ can be acquired whenever hydrogen bonding exists in the binding reaction [29,31]. Hence, it is not appropriate to justify the thermodynamic parameters according to a sole intermolecular force model (i.e., electrostatic); this needs to be further examined using the computational molecular simulation.

### 2.4. UV-Vis Absorption Spectra

**Figure 6:**
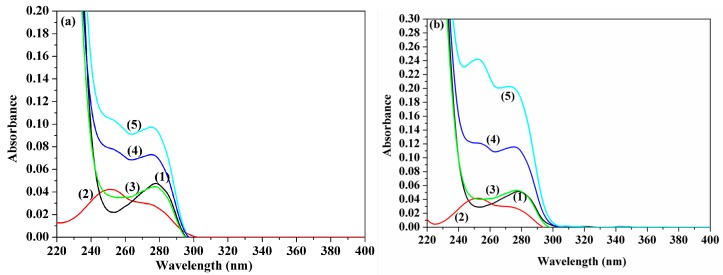
UV-Vis absorption determinations. Panel (**a**) shows ACV-HSA showing spectra of: (1) 1.5 μM HSA, (2) 7 μM ACV, (3) HSA subtracted from ACV complex, (4) 7 μM ACV + 1.5 μM HSA and (5) 15 μM ACV + 1.5 μM HSA. Panel (**b**) shows PNV-HSA showing spectra of: (1) 1.5 μM HSA, (2) 8 μM PNV, (3) HSA subtracted from PNV-complex, (4) 17 μM PNV + 1.5 μM HSA and (5) 35 μM PNV + 1.5 μM HSA.

The UV-Vis absorption spectral measurement represents another facile method used to investigate the structural changes and to detect the protein–drug complex formation. In the present study, the UV-Vis absorption spectra of HSA, in the presence and absence of ACV and PNV, were recorded at ambient temperature and are shown in Figure 6a,b. The presented spectra can further hypothesize the formation of new ACV-HSA/PNV-HSA complexes; this is supported by a steady increase in the peak intensity of ACV-HSA/PNV-HSA upon addition of higher ACV/PNV concentrations, with the observed change in the protein peak shape after subtracting the ligand peak, as shown in Figure 6a,b.

### 2.5. ACV/PNV Effect on HSA Conformation 

#### 2.5.1. Synchronous Fluorescence

**Figure 7:**
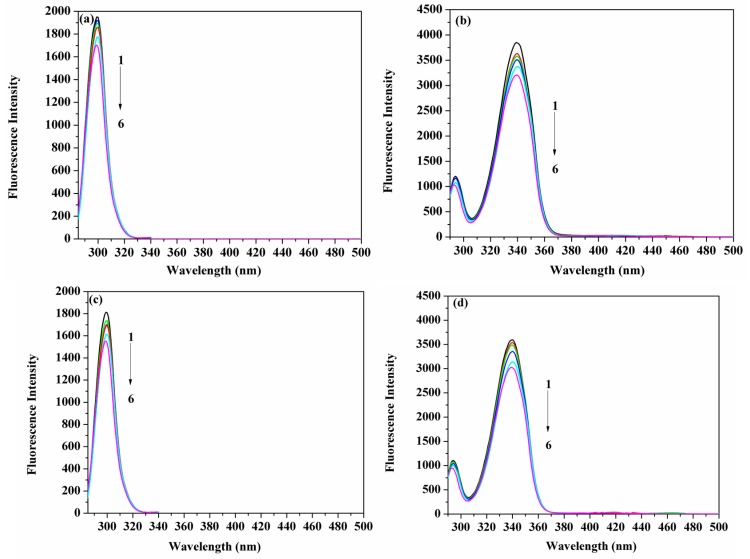
Spectra of the synchronous fluorescence of HSA (1.5 μM) with the addition of ACV (1–6 represent 0, 3.5, 7, 15, 25 and 35 μM, respectively) at Δλ = 15 nm (**a**) Δλ = 60 nm (**b**) and the addition of PNV (1–6 represent 0, 3.5, 8.0, 17, 28 and 35 μM, respectively) at Δλ = 15 nm (**c**) Δλ = 60 nm (**d**).

Observing the spectra resulting from synchronous fluorescence for the protein, in the presence and absence of ligands, can distinguish between the fluorescence emission arising from Tyr and Trp, when the measurements are performed at (Δλ) values of 15 nm and 60 nm, respectively [32,33,34]. Figure 7a–d demonstrate that the HSA fluorescence intensity was steadily reduced with the accumulation of ACV/PNV. It can be observed from those figures that no significant shift of the maximum emission wavelength occurred at Δλ = 15 nm (Figure 7a,c) or 60 nm (Figure 7b,d), either for ACV or PNV. Hence, it can be implied from Figure 7a–d that both ACV/PNV do not alter the conformation of HSA around the Tyr and Trp residues. 

#### 2.5.2. Three-Dimensional (3D) Fluorescence Measurements

**Figure 8:**
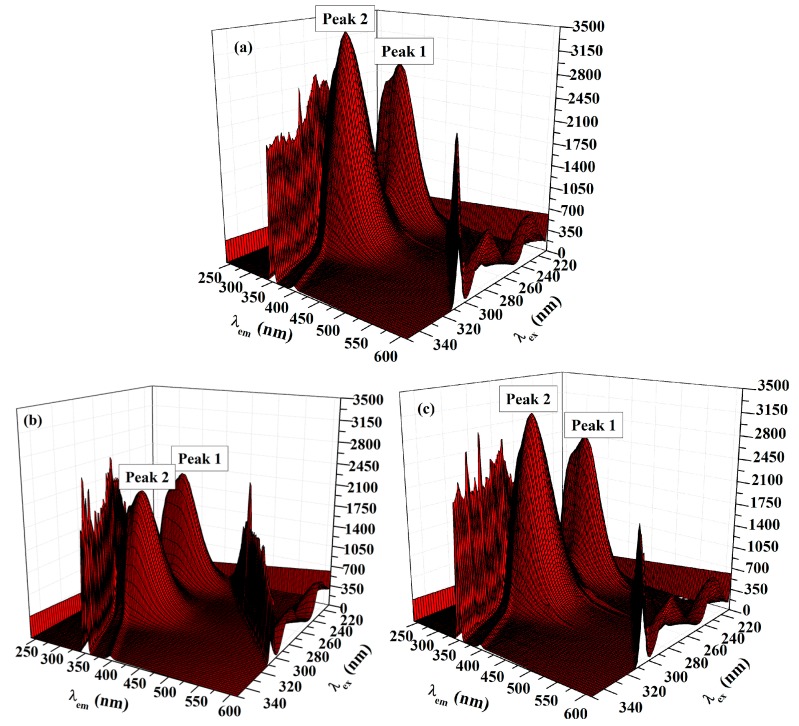
Three-dimensional (3D) spectra of HSA (1.5 μM) in the (**a**) absence and (**b**) presence of ACV (35 μM) and (**c**) in the presence of PNV (28 μM).

**Figure 9:**
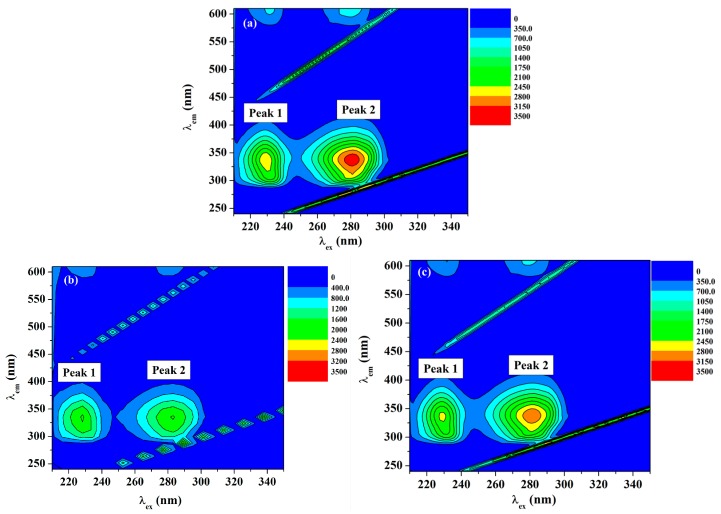
Contour plot of the fluorescence intensity spectra of (**a**) HSA, (**b**) ACV-HSA and (**c**) PNV-HSA systems.

Recording of the 3D fluorescence spectra was executed as an important approach to observe the changes in the protein conformation. Three-dimensional fluorescence spectra of HSA in the absence and presence of ACV/PNV were determined. Figure 8a–c and Figure 9a–c show the 3D surface and contour plots acquired for ACV/PNV-HSA and Table 3 summarizes the characteristic parameters obtained for such measurements. It can be observed from the figures (Figure 8a–c and Figure 9a–c) that HSA has two fluorescence peaks (1 and 2). Peak 1 was observed at λ_ex_ 228 and λ_em_ 340, while peak 2 was observed at λ_ex_ 280 and λ_em_ 340, with both peaks representing the spectral characteristics of Trp and Tyr residues that are principally due to the *n*→Π* transition of aromatic amino acids in HSA. Peak 2 demonstrates the spectral features of the polypeptide backbone (Π→Π* transition) [14]. Upon the addition of ACV/PNV to HSA, the fluorescence peaks of HSA were quenched with a no peak shift, as demonstrated in Figure 8a–c and Figure 9a–c, which may refer to changes in the microenvironment around Trp and Tyr residues. Figure 8 and Figure 9 also show a stronger quenching in the case of ACV, influencing both peaks, as summarized in Table 3. 

### 2.6. Site Markers Competitive Binding

**Figure 10:**
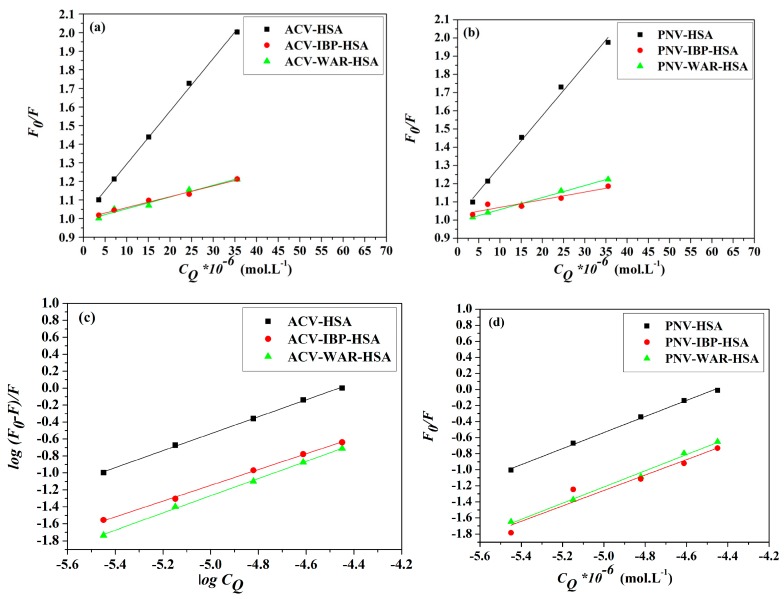
Stern–Volmer plots of (**a**) ACV (**b**) PNV and double log plots of (**c**) ACV and (**d**) PNV showing their interactions with HSA at 298 K, in the presence and absence of ibuprofen (IBP) and warfarin (WAR).

Several previous studies have established that HSA retains two principal ligand-binding sites, named Sudlow sites I and II, situated in subdomains IIA and IIIA, respectively [35,36,37]. Thus, it was imperative to systematically examine the binding sites for ACV/PNV on the HSA. This study used the binding of HSA to warfarin (WAR) as a marker for site I and ibuprofen (IBP) as a marker for site II. Following a steady increment of the ACV/PNV concentrations to solutions of HSA alone and HSA-bound site markers (1:1 ratio), intensities of the fluorescence emission were recorded at 298 K. Fluorescence data were used to plot graphs of the Stern–Volmer (Equation (1)) and double log correlations (Equation (4)), as presented in Figure 10a–d and subsequently the *K_sv_* and *K* values were calculated, as listed in Table 4. The results established that ACV-HSA/PNV-HSA binding in the presence of both site markers resulted in a significant diminution in *K_sv_* and *K* values, compared to ACV-HSA/PNV-HSA only complexes. Those findings hence assume that displacement interactions took place in both sites of the HSA, thus suggesting that both ACV and PNV may bind to HSA subdomains IIA and IIIA. 

### 2.7. Molecular Docking 

**Figure 11:**
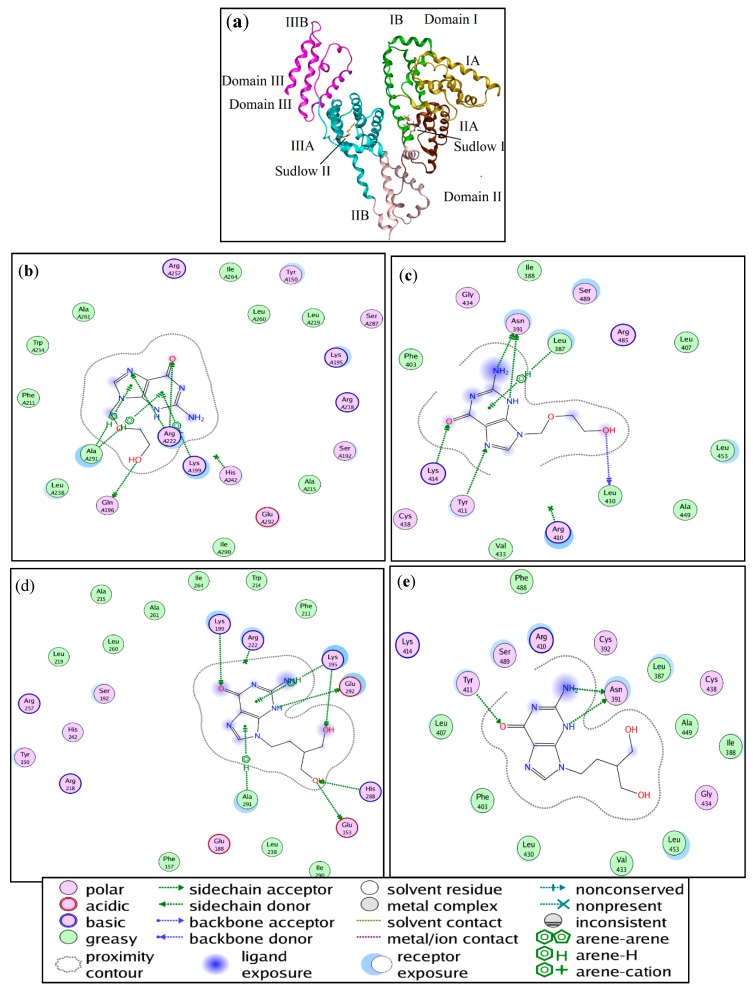
(**a**) A representation of the HSA subdomains and binding sites. Illustrations of the amino acid residues involved in the ACV-HSA interaction at (**b**) Sudlow site I and (**c**) Sudlow site II. Illustration of the amino acid residues involved in the PNV-HSA interaction at (**d**) Sudlow site I and (**e**) Sudlow site II.

Molecular docking has become one of the indispensable tools for drug discovery [38]. It correlates the proteins and ligands through the numerous forms of interactions. The molecular simulation results for the ACV/PNV-HSA binding, further demonstrate that both ACV and PNV can bind to both Sudlow sites I and II (subdomains IIA and IIIA) within the HSA structure (Figure 11a). Selection of the best configurations of ACV and PNV when bound to HSA revealed their lowest binding energy conformers, as shown in Figure 11b–e. Such conformers bind HSA with free energies of −25.61 and 22.01 kJ·mol^−1^ with root mean square deviation (RMSD) values of 2.26 and 1.11 for ACV, in HSA sites I and II, and −22.97 and −26.53 kJ·mol^−1^ with RMSD values of 0.76 and 1.19 for PNV, in HSA sites I and II. These bound ACV/PNV conformers are located among the active site residues listed in Table 5 and illustrated in Figure 11b–e. 

## 3. Materials and Methods 

### 3.1. Standards and Reagents

Unless otherwise stated, all standards, chemicals and reagents including ACV, PNV and HSA of highest purity (fatty acid free ~0.005%) were purchased from Sigma–Aldrich Co. (St. Louis, MO, USA). HPLC grade Methanol (MeOH) was obtained from BDH laboratory supplies (Poole, UK). All aqueous solutions utilized during the course of the experimental procedure were prepared using double distilled water procured from a Millipore Milli-Q^®^ UF-Plus purification system (Millipore, Bedford, MA, USA). 

### 3.2. Preparation of Experimental Solutions 

ACV and PNV stock solutions were prepared using HPLC-grade methanol in a concentration of 2.0 mM. Subsequent dilutions and all working solutions were prepared in a 1× phosphate buffered saline (PBS) (pH ~7.4). A stock solution of HSA (15 µM) was prepared in PBS, and subsequently diluted to a working solution of 1.5 µM. All experimental solutions were prepared at normal laboratory temperature and stored at −20 °C for long-term storage (over one week) and at 4 °C for daily use. 

### 3.3. Ligand-Induced Fluorescence Quenching of HSA 

The fluorimetric analyses of the ACV-HSA and PNV-HSA systems were performed with the aid of a Jasco FP-8200 (Jasco Int. Co. Ltd. Tokyo, Japan) using a 1 cm quartz cuvette. The emission spectra were monitored in the wavelength range 285–500 succeeding the analytes excitation at 280 nm with the slit widths set to 5 nm for excitation and emission. In order to examine the type of binding in the ACV/PNV-HSA interactions, fluorescence quenching determinations were carried out at three temperatures (288, 298 and 309 K). Measurements were performed using five aliquots of ACV/PNV in the concentration range of 3.5–35 μM individually mixed with the same volume of HSA 1.5 μM. The inner filter influence arising from the light absorbance by the compounds existing within the solution at the wavelengths of excitation/emission was minimized via the use of the following equation (Equation (7)) [19].

(7) $$F_{corr}{}_{=F_{obs}\;*\;antilog\frac{\left(A_{ex}+A_{em}\right)}{2}}$$

where *F_cor_* and *F_obs_* are the corrected and determined fluorescence intensities, respectively, while, *A_ex_* and *A_em_* are the ACV/PNV absorbance values measured at the same wavelengths of the protein excitation and emission, respectively.

### 3.4. Synchronous and 3D Fluorescence Measurements

Measurements of the synchronous fluorescence spectra of the ACV-HSA/PNV-HSA systems were carried out at ∆λ = 15 nm and 60 nm to reveal the tyrosine and tryptophan features of the HSA. In a similar vein, their 3D fluorescence spectra were recorded within the excitation wavelength range of 210–350 nm and emission wavelength range of 240–610 nm. 

### 3.5. Competitive Binding Studies

Binding displacement between ACV/PNV and the previously reported site markers, WAR and IBP, on the HSA was inspected with the aid of fluorometric measurements. WAR and IBP have been previously established as markers for the HSA binding sites, I and II, respectively. Site markers solutions were prepared using the same procedure described earlier to dissolve and dilute ACV and PNV. Experimental solutions of HSA and the site markers were kept at the concentrations of 1.5 μM concentration, while ACV/PNV concentrations were gradually varied between 3.5 and 35 μM.

### 3.6. UV-Vis Spectral Determinations

The UV-Vis spectra of the ACV-HSA/PNV-HSA mixtures were monitored over a wavelength range of 220–400 nm using a double beam UV-Vis spectrophotometer (UV-1800 Schimadzu^TM^, Schimadzu Corporation, Tokyo, Japan). Measurements were executed using a 1.5 μM HSA concentration as sole solution and following its binding to 7.0 and 15 μM concentrations of ACV and 3.5, 17 and 35 concentrations of PNV, while using solutions of 7.0 μM and 8.0 for individual ligand measurements. 

### 3.7. Molecular Docking 

The HSA three-dimensional structure (PDB 1E78) was acquired from the Protein Data Bank and uploaded into the Molecular Operating Environment software package (MOE^®^ 2014, Chemical computing group, Montreal, QC, Canada) for pre-adjustment through water molecules/heteroatom removal and hydrogen atom addition. The ligands’ 3D structures were graphed using ChemDraw^®^ Ultra 14.0 software (Cambridge Soft, Cambridge, MA, USA); minimized energy structure and geometries of ACV/PNV were obtained by MOE^®^ 2014 software package (Chemical computing group, Montreal, QC, Canada) in the compatible file format. The binding pockets on the HSA were selected and the London dG scoring function and the rescoring function GBVI/WSA dG in MOE^®^ were set to sort the docked postures of the ACV/PNV. The best conformers of ACV/PNV with HSA were nominated based on the scoring and RMSD (root mean square deviation) values. 

## 4. Conclusions

The interactions of the antiviral drugs, ACV and PNV, with the HSA were explored by means of different spectroscopic tools with the determination of their binding parameters. The study established that ACV/PNV statically quench the fluorescence intensity of HSA via the development of non-fluorescent complexes with association constants (*K_b_*) of 2.89 ± 0.01and 2.68 ± 0.02 × 10^4^ M^−1^ for ACV and PNV, respectively. Values of the Stern–Volmer (*K_sv_*) and the binding constants (*k*) exhibited that ACV/PNV bind HSA with high affinity and the negative *ΔG^0^* value indicated their spontaneous reactions. The HSA interaction with both ACV and PNV was shown to be exothermic, owing to the computed negative value of Δ*H^0^*. Binding of ACV/PNV to HSA with the existence of formerly described site markers (IBP and WAR) proved that both ACV and PNV can bind to Sudlow sites I and II of the HSA. The latter conclusion was further verified by molecularly simulating the interactions of ACV/PNV to HSA, which presented the most stable configuration of the ACV/PNV-HSA systems and located the best ACV and PNV conformers within sites I and II of the HSA. Such data acquired from this study may be of significant importance when employed to further comprehend the pharmacokinetic profiles of these two widely used antiviral agents.

## Figures and Tables

**Table 1:** Parameters computed from the Stern–Volmer and Lineweaver–Burk relations for ACV and PNV interactions with HSA.

Ligand	Measured Parameters		Temperature (K)		
			288	298	309
**ACV**	**Stern-Volmer parameters:**	***K_SV_* × 10^4^ (M^−1^)**	2.96 ± 0.06	2.84 ± 0.07	2.75 ± 0.07
		***K_q_* × 10^13^ (M^−1^ ·s^−1^)**	1.10	1.05	1.02
		***R^2^***	0.9987	0.9984	0.9978
	**Lineweaver–Burk parameters:**	***K_LB_* × 10^4^ (M^−1^)**	2.76 ± 0.11	2.64 ± 0.14	2.56 ± 0.06
		***R*^2^**	0.9988	0.9991	0.9999
**PNV**	**Stern-Volmer parameters:**	***K_SV_* × 10^4^ (M^−1^)**	3.04 ± 0.15	2.77 ± 0.09	2.60 ± 0.13
		***K_q_* × 10^13^ (M^−1^· s^−1^)**	1.13	1.03	0.96
		***R*^2^**	0.9922	0.9964	0.9918
	**Lineweaver–Burk parameters:**	***K_LB_ ×* 10^4^ (M^−1^)**	3.64 ± 0.18	3.13 ± 0.16	2.80 ± 0.14
		***R*^2^**	0.9941	0.9978	0.9986

*K_SV_*: Stern-Volmer constant; *K_q_*: bimolecular quenching rate constant.

**Table 2:** Thermodynamic parameters and binding data for the ACV/PNV-HSA interactions.

	Temp. (K)	∆*G*^0^(kJ·mol^−1^)	∆*H*^0^ (kJ·mol^−1^)	∆*S*^0^ (J·mol^−1^·K^−1^)	*K* × 10^4^ (L·mol^−1^)	*n* *	*R*^2^
**ACV**	288	−24.66 ± 0.02	−1.79 ± 0.29	79.40 ± 0.95	2.97 ± 0.02	1.00 ± 0.04	0.9991
	298	−25.45 ± 0.01			2.89 ± 0.01	1.00 ± 0.02	0.9992
	309	−26.33 ± 0.01			2.82 ± 0.01	1.00 ± 0.01	0.9994
**PNV**	288	−24.62 ± 0.03	−4.47 ± 0.51	69.95 ± 1.69	2.99 ± 0.03	1.01 ± 0.05	0.9936
	298	−25.32 ± 0.02			2.68 ± 0.02	1.00 ± 0.03	0.9979
	309	−26.08 ± 0.02			2.59 ± 0.03	1.00 ± 0.03	0.9973

* All values are average of three determinations. Note, Temp.: temperature; ∆*G*^0^: Gibbs free energy; ∆*H*^0^: enthalpy change; ∆*S*^0^: entropy change; *K*: binding constant; *n*: number of binding sites; R^2^: correlation coefficient.

**Table 3:** Data obtained from 3D fluorescence experiments for HSA in presence and absence of ACV and PNV.

	Relative Intensity (IF)	Relative Intensity (IF)
	1st Peak 228/340 (λ_ex_/λ_em_) nm	2nd Peak 280/340 (λ_ex_/λ_em_) nm
**HSA**	2726.96	3370.78
**ACV-HSA**	2063.92 (↓663.04; 24.31%)	2042.05 (↓1328.73; 39.42%)
**PNV-HSA**	2551.82 (↓175.14; 6.42%)	3079.69 (↓291.09; 8.64%)

**Table 4:** Estimated Stern–Volmer constants for ACV/PNV with HSA, in the presence and absence of site markers.

Systems	*K_SV_* × 10^4^ (L·mol^−1^)	*R^2^*	*K* × 10^4^ (L·mol^−1^)	*R^2^*
**ACV-HSA**	2.84 ± 0.07	0.9984	2.89 ± 0.01	0.9992
**ACV-HSA + IBP**	0.58 ± 0.03	0.9947	0.32 ± 0.05	0.9988
**ACV-HSA + WAR**	0.63 ± 0.06	0.9863	0.48 ± 0.03	0.9989
**PNV-HSA**	2.77 ± 0.09	0.9964	2.68 ± 0.02	0.9979
**PNV-HSA + IBP**	0.42 ± 0.08	0.9463	0.35 ± 0.06	0.9457
**PNV-HSA + WAR**	0.66 ± 0.02	0.9972	0.67 ± 0.02	0.9938

**Table 5:** Molecular simulation results for ACV/PNV interactions with HAS.

Ligand	Binding Site	Amino Acid Residues	Interaction Type	Distance (Å)	Total Binding Energy (kJ·mol^−1^)
**ACV**	Site I	Gln196	H-donor	2.87	−25.61
		Lys199	cation-π	3.80	
		Arg222	H-acceptor	3.15	
		Arg222	H-acceptor	3.06	
		Ala291	H-π	4.19	
		Ala291	H-π	4.37	
	Site II	Leu387	H-π	4.43	−22.01
		Asn391	H-donor	3.01	
		Tyr411	H-acceptor	3.36	
		Lys414	H-acceptor	2.85	
		Leu430	H-donor	3.12	
**PNV**	Site I	Glu153	H-donor	3.14	−22.97
		Lys195	H-π	4.05	
		Lys195	H-acceptor	3.22	
		Lys199	H-acceptor	3.18	
		His288	H-acceptor	3.12	
		Ala291	H-π+	4.26	
		Glu292	H-donor	2.82	
	Site II	Asn391	H-donor	3.22	−26.53
		Asn391	H-donor	2.96	
		Tyr411	H-acceptor	2.87	
